# Beyond the Surface: Antinuclear Antibodies in Rheumatoid Arthritis—Experiences from a Single-Center, Cross-Sectional Observational Study

**DOI:** 10.3390/antib15040058

**Published:** 2026-07-10

**Authors:** Hanna Cholerzyńska, Gabriela Kot, Łukasz Świątek, Bogna Grygiel-Górniak

**Affiliations:** 1 Doctoral School, Poznan University of Medical Sciences, 60-812 Poznań, Poland; 2 Division of Rheumatology and Clinical Immunology, Beth Israel Deaconess Medical Center, Harvard Medical School, Boston, MA 02215, USA; 3 Department of Rheumatology, Rheumatological Centre, 63-100 Śrem, Poland; 4 Department of Rheumatology, Rehabilitation and Internal Diseases, Poznan University of Medical Sciences, 61-545 Poznań, Poland

**Keywords:** antinuclear antibodies, rheumatoid arthritis, diagnosis, co-morbidities

## Abstract

**Background:** Antinuclear antibodies (ANA) can be detected in patients with rheumatoid arthritis (RA) and pose many diagnostic challenges, especially when RA presents an atypical course and requires differentiation from other systemic connective tissue diseases (sCTDs). This study assessed ANA fluorescence patterns and immunoblot profiles, as well as the relationships between ANA titers, antibody expression intensity, and markers of disease activity in patients with RA. **Methods**: This single-center, cross-sectional, observational study included 81 RA patients (53 ANA-positive) meeting the 2010 ACR/EULAR classification criteria. ANA titers and fluorescence patterns were assessed using indirect immunofluorescence. Anti-extractable nuclear antigen (ENA) autoantibody profiles and expression intensity were assessed using immunoblot analysis. Demographic, clinical, and laboratory data were obtained. Spearman’s rank correlation coefficient was used to analyze the relationship between ANA titers and selected variables. Univariate and multivariate logistic regression analyses were performed to identify factors associated with ANA positivity. **Results**: The cohort consisted primarily of women (86.4%) with moderate disease activity. ANA fluorescence patterns were heterogeneous, with nucleolar and homogeneous patterns most frequently observed. Immunoblot analysis also revealed diverse autoantibody profiles without a clearly dominant specificity. Ro-52, SS-A, and Sm antibodies were detected more frequently, although their prevalence remained relatively low. No statistically significant correlations were found between ANA titers and inflammatory markers, serological parameters, or disease activity indices. **Conclusions**: RA patients with positive ANA demonstrated marked immunological heterogeneity, without concomitant symptoms of sCTD. A positive result in RA may reflect generalized immune dysregulation rather than a distinct clinical subtype. Further studies with larger cohorts are needed to clarify the clinical significance of ANAs in rheumatoid arthritis.

## 1. Introduction

Serological testing is an essential part of sCTD diagnostics [1]. In RA, the most recognized and clinically confirmed serological markers are rheumatoid factor (RF) and autoantibodies against cyclic citrullinated peptide (aCCP) [2]. In addition to conventional RA-associated markers, ANAs can also be detected in some patients [3]. These antibodies constitute a diverse group of autoantibodies directed against intracellular nuclear proteins, often associated with sCTD, such as systemic lupus erythematosus, systemic sclerosis, Sjögren’s syndrome, mixed connective tissue disease, and many others [4]. Studies indicate that a positive ANA test result may occur in approximately 40% of patients with RA, suggesting a more extensive immune dysregulation beyond the classic RA serological profile [3]. ANA antibodies can be found in patients with dermatitis, immune deficiencies, chronic bacterial or viral infections, and malignancies. These conditions may produce false-positive results [5].

However, the clinical significance of ANA in RA remains unclear. Available data remain inconsistent, and the significance of specific ANA fluorescence patterns, ANA titers, and antibody immunoblots in RA has not been definitively established [6]. Determining which ANAs may appear in RA and whether they correlate with disease activity should provide answers to these puzzling questions. Therefore, the aim of this study was to determine the clinical, laboratory, and immunological characteristics of ANA-positive RA patients, with particular emphasis on titers, fluorescence patterns, and antibody profiles, as well as potential associations with selected markers of inflammation and disease activity.

## 2. Materials and Methods

### 2.1. Protocol of the Study

**Figure 1:**
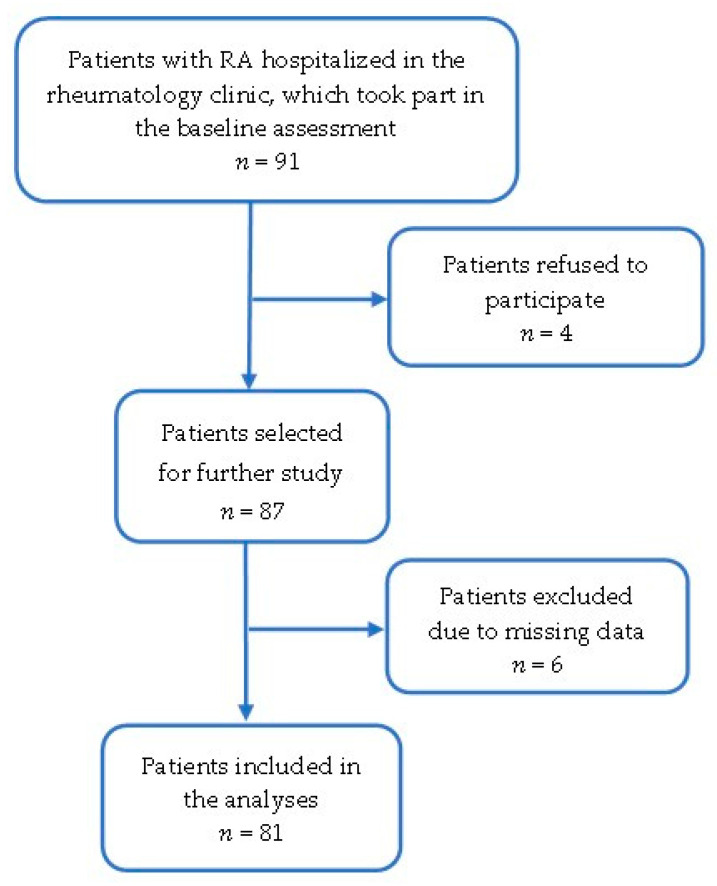
Flow chart of patients’ selection.

This single-center, cross-sectional, observational study included 81 patients diagnosed with RA according to the 2010 ACR/EULAR classification criteria and treated at the rheumatology department of a clinical hospital in Poland [7,8,9]. The study was conducted between 2023 and 2024 (Figure 1). Eligible patients were 18 years of age or older, had RA for at least one year (diagnosis confirmed by two rheumatologists at disease onset and on the day of study enrollment), and were willing to participate. Patients with other or overlapping connective tissue diseases, malignancies, severe liver or kidney disease, infection (including active or chronic viral, bacterial, and fungal infections), and incomplete clinical or laboratory records were excluded. These diseases or health problems may cause inflammation, leading to false-positive results. The study was conducted in accordance with the ethical principles of the Declaration of Helsinki. All participants provided informed consent. The study protocol was approved by the Bioethics Committee of the Poznań University of Medical Sciences, KB-854/23.

### 2.2. Anthropometric Measurements

Patients were measured after an overnight fast, in underwear and without shoes. Anthropometric assessments included height and weight. Weight and height were measured using an OMRON BF511 scale. Body weight was measured to the nearest 0.1 kg, and height was estimated using a vertical ruler to the nearest 1 mm. Body mass index (BMI) was calculated as body weight (kg) divided by the square of height (m^2^).

### 2.3. Rheumatoid Arthritis Activity

RA activity was assessed using the DAS28 (ESR). The index value is calculated based on the number of tender (TEN28) and swollen (SW28) joints, the erythrocyte sedimentation rate (ESR) and the patient’s assessment of the disease (Visual Analog Scale—VAS), according to the formula: DAS28 = 0.56 √ (TEN28) + 0.28 √ (SW28) + 0.70 Ln (ESR) + 0.014 (VAS) [10]. The DAS28 (ESR) has been extensively validated for its use in clinical trials in combination with the EULAR response criteria [8,9].

### 2.4. Biochemical Analysis

Blood samples were collected between 7:00 and 8:00 AM after an overnight fast. Venous blood samples were collected in EDTA or heparin tubes and immediately centrifuged. Enzymatic colorimetric assays were used to determine erythrocyte sedimentation rate (ESR) and C-reactive protein (CRP). CRP was measured using an Abbott Architect C16000 instrument. ESR was measured using the Halifax Test 1 device from Biomedica.

### 2.5. Serological Analysis

In our patients, these ANA analyses were performed to assess their titer, fluorescent pattern, and profile in patients diagnosed with RA. Clinical and serological evaluation allowed for the inclusion of patients without other connective tissue diseases (sCTDs) or overlap syndromes.

Qualitative and quantitative antibody assessment was performed by indirect immunofluorescence (IIF). This method enabled estimation of ANA titers and nuclear fluorescence patterns using human epithelial HEp-2 cells and primate liver cells. These cells express a broad spectrum of human nuclear antigens and enable evaluation of fluorescence patterns. Fluorescence patterns were interpreted by experienced laboratory personnel and classified according to the International Consensus on ANA Patterns (ICAP) recommendations: homogeneous, nucleolar, speckled, or centromeric.

ANA titers were determined using several serum dilutions. Samples were initially screened at a 1:40 dilution and, if positive, were further diluted to determine final titers ranging from 1:80 to 1:5120. ANA titers of 1:40–1:80 were classified as weakly positive, titers of 1:160–1:640 as moderately positive, and titers ≥1:1280 as strongly positive. All analyses were performed according to routine laboratory quality control procedures.

Serum antibodies against specific antigens were detected using standard immunoblot protocols. Immunoblot bands were analyzed using the EUROLineScan software (EUROIMMUN, Lübeck, Germany, Version 3.4.37), according to the manufacturer’s instructions. This method allows for the evaluation of the following antibodies: anti-RNP/Sm—anti-ribonucleoprotein/Sm complex; anti-Sm—Smith’s antigen; anti-SS-A—native Sjögren’s syndrome antigen A (Ro 60 kDa autoantigen); anti-Ro-52—Sjögren’s syndrome antigen A (Ro 52 kDa autoantigen); anti-SS-B—Sjögren’s syndrome antigen B (La autoantigen); anti-Scl-70—systemic sclerosis antigen; anti-PM-Scl100—polymyositis/systemic sclerosis/overlap syndromes antigen 100 antibodies; anti-Jo-1—histidyl-tRNA synthetase antigen; anti-Centromere B; anti-PCNA—proliferating cell nuclear antigen; anti-dsDNA—double-stranded DNA antigen; Nu—nucleosome antigen; Hi—histone antigen; anti-rPP—ribosomal protein P antigen; anti-DFS70—Dense Fine Speckled antigen, molecular weight 70 kDa. Positive and negative controls were included in each analytical run, as recommended by the manufacturer. Antibody reactivity was expressed as light intensity (LI). Results were classified as negative (LI 0–7), borderline (LI 8–14), positive (LI 15–70), and strongly positive (LI 71–255), according to the manufacturer’s validated interpretation criteria.

IgG anti-CCP antibodies were analyzed using an ELISA enzyme-linked immunosorbent assay on the EUROIMMUN ANALYZER I-2P (Euroimmun, Lübeck, Germany; values >5 RU/mL were considered positive; software Version 1.11.3). Rheumatoid factor was analyzed by an immunoturbidimetric method (TIA), and values >14 U/mL were considered positive.

### 2.6. Statistical Analysis

Patient characteristics were summarized using standard descriptive statistics for continuous and categorical variables (demographic and clinical data). Continuous variables were assessed for normality using the Shapiro–Wilk test. Because most parameters were not normally distributed, data were presented as medians and interquartile ranges (IQR). Categorical variables were presented as counts and percentages. Spearman’s rank correlation coefficient was used to determine the association between ANA titers and selected inflammatory and serological variables. Univariate analysis was performed to determine the association between ANA positivity in RA and demographic, clinical, and laboratory variables. Each variable was tested individually to determine its odds ratio and confidence interval.

After univariate analysis, variables with *p* < 0.25 were included in a multivariate logistic regression model, following the recommendations of Hosmer and Lemeshow, who suggested using a more liberal significance threshold during preliminary variable selection to avoid excluding potentially important predictors [11]. To account for potential interactions between variables, the correlation matrix was analyzed. No significant multicollinearity was observed. Statistical analysis was performed using Statistica software (version 13.1, TIBCO Software Inc., Palo Alto, CA, USA). All tests were two-sided, and *p*-values < 0.05 were considered statistically significant.

## 3. Results

The patient’s characteristics are present in Table 1. RA had been diagnosed approximately 14 years previously (median), and the disease duration ranged from 9 to 24 years (interquartile range). The tests showed elevated ESR and CRP values, with a DAS28 index of 4.18 (3.28–5.10), indicating moderate RA activity. Antirheumatic treatment patterns according to ANA status are presented in Supplementary Table S1. Methotrexate was the most commonly used cDMARD in both groups, followed by biologic agents and corticosteroids. Overall, treatment distributions were comparable between groups (all *p* > 0.05).

Among ANA-positive RA patients, the most frequently observed titers were moderately positive (Table 2). Furthermore, ANA fluorescence patterns showed significant heterogeneity, with nucleolar and homogeneous patterns being the most common.

Immunoblot assays also revealed a broad spectrum of autoantibody specificities without a dominant profile. Among the antibodies detected, Ro-52, SS-A, and Sm were most frequently observed, although their overall prevalence remained low (Table 2 and Table 3).

Spearman correlation analysis revealed no statistically significant associations between ANA titers, inflammatory and serological markers, or disease activity parameters in patients with RA. A weak positive correlation was observed between ANA titers and anti-CCP antibody levels, but this association was statistically insignificant (*p* > 0.05) (Table 4).

Univariate logistic regression analysis was performed using selected demographic, clinical, and laboratory variables to assess factors associated with ANA positivity in patients with RA (Table 5). None of the parameters considered were significantly associated with a positive ANA test result. However, some trends were observed, indicating weak, statistically insignificant associations between ANA results and current smoking (the highest odds ratio) and disease duration, anti-CCP antibody levels, ESR, and the number of tender joints (*p* < 0.25).

Given the potential to stimulate ANA synthesis in active disease, we examined whether clinical and serological activity in RA could influence ANA synthesis. Variables with *p*-values less than 0.25 in the baseline comparisons (Table 5), including disease duration, CCP, and number of swollen joints, were also included in the multivariate logistic regression analysis (Table 6). To minimize the risk of multicollinearity, correlations between independent variables were assessed before multivariate analysis. No significant associations were identified, suggesting that a positive antinuclear antibody test occurred independently of the factors analyzed.

## 4. Discussion

For many years, there has been debate over whether ANAs in RA are clinically significant or merely a consequence of nonspecific disease activity. These antibodies are routinely measured in clinical practice in RA because they are not specific for this disease. Instead, they are performed at the onset of developing RA when the arthritis has an atypical course, symptoms are mild, or another sCTD is suspected [12,13]. To address the numerous clinical uncertainties regarding ANA synthesis in RA, we analyzed the presence of these antibodies (titers, fluorescence type, and profile) in the context of clinical, laboratory, and immunological features in our RA patient cohort.

The median age of our patients was 58 years, and 86.4% were women. This distribution is typical of RA, with 70–80% of cases in women [14,15]. A total of 14.8% of patients smoked cigarettes, 32.1% were former smokers, and 53.1% of patients reported having never smoked. Cigarette smoking is known to be the strongest environmental risk factor for RA. Studies have shown that in individuals with positive anti-citrullinated protein (anti-CCP) antibodies, an interaction between the common epitope (SE) and smoking occurs, which increases the risk of RA [16,17,18]. In our patients, positive aCCP values were detected in 68 (81.42%) and RF in 79 (97.14%) patients.

In our study, the majority of patients were ANA positive (65.4%), which is higher than the ANA prevalence reported in other studies, which range from 25 to 40% [19,20,21]. Several factors may explain this discrepancy. First, our cohort consisted exclusively of hospitalized RA patients, with a median disease duration of 14 years, potentially reflecting a population with greater cumulative immune dysregulation than in outpatient cohorts. Although the relationship between ANA positivity and classical autoantibodies in RA is not fully understood, it is possible that immune overstimulation contributes to the synthesis of both RF and aCCP, as well as the higher prevalence of ANAs. Finally, ANA was assessed by IIF at an initial screening dilution of 1:40, which may increase sensitivity compared with studies using more restrictive cutoff values.

Based on ANA titer, patients were classified as positive (*n* = 53) or negative (*n* = 28). Patients with a positive ANA result were divided into three groups: weakly positive (*n* = 9), moderately positive (*n* = 37), and strongly positive (*n* = 7). In our study, we determined ANA titer (which ranged from weak to strongly positive) and staining patterns. It is well known that ANA fluorescence patterns are associated with specific sCTDs [22]; however, previous studies have not clearly demonstrated any specific ANA pattern in patients with RA [23]. In our study, nucleolar and homogeneous patterns were most frequently observed, but the overall distribution of fluorescence patterns remained heterogeneous. On the other hand, Nakano et al. noted a clear predominance of speckled and homogeneous ANA patterns in patients with RA [19]. Similarly, Liu et al. demonstrated that homogeneous and speckled ANA patterns were most common in the RA population [20]. However, available data remain very limited, as current guidelines do not mandate ANA testing in every RA patient.

The literature emphasizes that ANA fluorescence patterns are characterized by relatively low sensitivity and specificity for individual sCTDs and are therefore currently being replaced by immunoblot analysis [3]. Therefore, in our study, immunoblot analysis was performed to identify individual ANAs. It revealed significant heterogeneity in ANA profiles (Table 3). Similar ANA heterogeneity was observed in the study by Emad et al. [24]. These results may support the hypothesis that antibodies in the ANA profile in RA reflect only generalized B-cell activation and immune dysregulation [25,26].

The pathogenesis of RA is characterized by impaired B-cell tolerance checkpoints, the expansion of autoreactive B-cell clones, and sustained B-cell activation. Chronic inflammatory stimulation, driven by cytokines such as BAFF (B-cell activating factor), IL-6, and TNF-α, promotes B-cell survival and differentiation into plasma cells. Such abnormalities may promote the production of a broad spectrum of autoantibodies beyond the classical markers of RA [27]. Therefore, the heterogeneity of ANA fluorescence patterns and ENA profiles observed in our cohort may reflect nonspecific polyclonal B cell activation rather than the presence of a distinct connective tissue disease (CTD) with an overlapping clinical presentation.

Interestingly, a recent study by Akar et al. showed that ANA-positive RA patients were more likely to require biologic therapy and treatment escalation than ANA-negative patients, despite comparable baseline characteristics. The authors suggested that ANA positivity may identify a subgroup of patients with distinct immunologic characteristics. Although treatment outcomes were beyond the scope of our study, these results further support the notion that ANA positivity may reflect broader immune dysregulation [28].

In our study of patients with RA, the most frequently detected antibodies by immunoblot were anti-SSA (anti-Ro60), anti-Ro52, and anti-Sm. In addition, anti-Ro52 antibodies showed the highest expression intensity, with the greatest number of strongly positive results (Table 3). A study by Schneeberger et al. showed that anti-Ro 52/60 antibodies in patients with RA are not clinically significant; in contrast, Ma et al. demonstrated that the isolated presence of anti-Ro52 antibodies may define a distinct serological subgroup of patients with RA [29,30]. In their study group, patients with isolated anti-Ro52 antibodies exhibited higher rheumatoid factor levels and a more pronounced autoimmune profile than seronegative individuals [30,31]. Anti-Ro52 antibodies (together with anti-SSA/Ro60 kDA antibodies) occur in Sjögren’s syndrome and systemic lupus erythematosus (particularly subacute cutaneous lupus erythematosus and neonatal lupus erythematosus) [31]. It is also worth emphasizing that RF occurs in both diseases, with a prevalence of approximately 75–95% in primary Sjögren’s syndrome and 15–35% in SLE, depending on the study population and the test used [32,33]. Anti-Sm antibodies are highly specific for SLE and are considered one of the immunological diagnostic criteria for this disease [34,35]. The detection of ENA antibodies, typically associated with CTD, may have potential clinical implications. Although the presence of these autoantibodies alone is not sufficient to make a diagnosis of CTD, a positive ANA test result may precede the development of clinical symptoms by several years [35]. Therefore, RA patients who have a positive ANA test result and are reactive to ENA may benefit from regular reassessment, especially if new symptoms suggestive of CTD develop [12,31].

Another important finding of this study was the lack of statistically significant correlations between ANA titers and inflammatory markers, serological parameters, or disease activity. Similarly, in the study by Paknikar et al., ANA positivity in RA did not correlate with radiographic changes or treatment intensity [21]. However, the lack of statistical significance in our study should be interpreted with caution, as the relatively small sample size may have limited the statistical power to detect moderate effects. Therefore, we cannot exclude the possibility that clinically relevant associations exist but remain undetected in the present cohort.

In this study, neither univariate nor multivariate logistic regression analysis identified significant predictors of ANA positivity in patients with RA. Although modest trends were observed for disease duration, anti-CCP antibody levels, and the number of swollen joints, none of these associations reached statistical significance. This supports the notion that ANA positivity in RA can occur independently of the classic inflammatory and serological features of this disease.

## 5. Limitations and Benefits of the Study

Several limitations of this analysis should be noted. The main limitations were the relatively small sample size, which may impact the reliability of the results, particularly the stability of the multivariate logistic regression model, and the risk of model overfitting. Further studies with larger numbers of participants are needed to support more convincing conclusions and enable reliable analyses of the associations between specific ANA fluorescence patterns, ENA antibody profiles, and serological markers. The single-center design is a potential limitation because the study population is relatively homogeneous, which may reduce the comparability and generalizability of the results. Therefore, further large-scale multicenter studies, stratified by ethnicity and genetic background, are needed to confirm the present results.

Furthermore, the cross-sectional study design did not allow for assessing changes in ANA profiles over time. The study lacks detailed data regarding additional autoimmune comorbidities, including autoimmune thyroid disease or diabetes, which could potentially influence ANA positivity. Therefore, the potential confounding effect of co-existing autoimmune diseases on ANA prevalence could not be fully assessed. Another limitation is the lack of an external comparison group. Consequently, the specificity of the observed ANA fluorescence profiles for RA could not be directly assessed. Future studies with a control group (healthy patients) would help to better define the clinical and immunological significance of ANA positivity in RA. Moreover, although treatment data were available and presented in Supplementary Table S1, the relatively small sample size and frequent use of combination therapy limited our ability to assess the independent effects of specific DMARDs (conventional and biological) and corticosteroids on ANA positivity and ENA antibody profiles.

Despite these limitations, the present study has several important strengths. To our knowledge, relatively few studies have examined both ANA fluorescence patterns and ENA intensity profiles in detail by immunoblot analysis in patients with RA. Combining indirect immunofluorescence and immunoblot analysis allowed for a more comprehensive immunological characterization of ANA-positive RA patients. Furthermore, the study highlights the significant heterogeneity of the ANA-related immune response in RA and supports the notion that ANA positivity in these patients may primarily reflect generalized immune dysregulation.

## 6. Conclusions

In ANA-positive RA patients, significant heterogeneity in both ANA fluorescence patterns and ENA profiles was observed, without identifying a disease-specific phenotype. No significant correlations were observed between ANA titers and inflammatory markers, disease activity, or classical serological parameters. ANA positivity in RA may be associated with broader immune dysfunction and polyclonal B-cell activation rather than a distinct overlap syndrome or specific clinical subtype.

## Figures and Tables

**Table 1:** Characteristics of patients with rheumatoid arthritis.

	Patients with Rheumatoid Arthritis			
Analyzed Parameters	ANA-Positive Patients (*n* = 53)	ANA-Negative Patients (*n* = 28)	All Patients (*n* = 81)	*p*-Value
	*n* (%)	*n* (%)	*n* (%)	
Female, *n* [%]	47 (88.7)	23 (82.14%)	70 (86.42)	0.4141
Place of residence				0.7899
Village	18 (64.29%)	10 (35.71%)	28 (34.57%)	
Town < 50,000 citizens	15 (71.43%)	6 (28.57%)	21 (25.93%)	
Town ≥ 50,000 citizens	20 (62.50%)	12 (37.5%)	32 (39.51%)	
Marital status				0.4464
Single	4 (66.67%)	2 (33.33%)	6 (7.41%)	
Divorced	6 (60%)	4 (40%)	10 (12.35%)	
Widower	3 (60%)	2 (40%)	5 (6.17%)	
Married	37 (71.15%)	15 (28.85%)	52 (64.20%)	
Partnership	3 (37.5%)	5 (62.5%)	8 (9.88%)	
Number of antirheumatic medications				0.2931
0	1 (50%)	1 (50%)	2 (2.47%)	
1	17 (54.84%)	14 (45.16%)	31 (38.27%)	
2	24 (77.42%)	7 (22.58%)	31 (38.27%)	
≥3	11 (64.71%)	6 (35.29%)	17 (20.99%)	
	Me + IQR (Q1–Q3)	Me + IQR (Q1–Q3)	Me + IQR (Q1–Q3)	
Age [years]	57 (45–67)	59 (53–69)	58 (47–68)	0.3455
Duration of the disease [years]	15 (10–24)	12 (8–17)	14 (9–24)	0.1721
Smoking status				0.1930
Current	7 (58.33%)	5 (41.67%)	12 (14.81%)	
Past	14 (53.85%)	12 (46.15%)	26 (32.10%)	
Never smoked	32 (74.42%)	11 (25.58%)	43 (53.09%)	
BMI [kg/m^2^]	23.63 (22.66–28.07)	24.96 (22.07–31)	24.89 (22.27–29.22)	0.5917
Swollen joint count	3 (1–6)	3 (1–4.5)	3 (1–6)	0.3532
Tender joint count	8 (6–14)	7 (5.5–10.5)	7 (6–12)	0.3986
CRP [mg/L]	8.8 (2.9–12)	4.8 (2.6–12.15)	5.6 (2.6–12)	0.4042
ESR [mm/h]	20 (14–34)	15.5 (10–34)	19 (12–34)	0.1866
DAS28 (ESR)	4.21 (3.49–5.26)	3.89 (3.19–4.81)	4.18 (3.28–5.10)	0.2452
RF [IU/mL]	120 (65.8–162.3)	107.9 (49.5–166.4)	120 (65.8–162.3)	0.9960
RF-positive [*n*] (%)	52 (98.11%)	27 (96.43%)	79 (97.14%)	
Anti-CCP [RU/mL]	89.9 (23.5–134.4)	77.8 (44.3–109.4)	87.6 (34.1–123.2)	0.5222
Anti-CCP [*n*] (%)	43 (81.13%)	25 (89.29%)	68 (81.42%)	

IQR—Interquartile range, BMI—body mass index, CRP—C-reactive protein, ESR—erythrocyte sedimentation rate, DAS28—disease activity score, RF—rheumatoid factor, Anti-CCP—anti-cyclic citrullinated peptide autoantibodies.

**Table 2:** Fluorescence patterns and titers of antinuclear antibody profiles in RA patients.

	Antinuclear Antibodies in RA Patients (*n* = 53)		
ANA	Type of Fluorescence		*n* (%)
ANA pattern		Nucleolar	20 (37.74%)
		Homogenous	16 (30.19%)
		Centromere	12 (22.64%)
		Speckled	5 (9.43%)
ANA titer		Weakly positive (1:40–1:80)	9 (17%)
		Moderately positive (1:160–1:640)	37 (69.8%)
		Strongly positive (≥1:1280)	7 (13.2%)

**Table 3:** The presence of selected antibodies in ANA-positive RA patients.

	Luminous Intensity of Selected ANA (*n* = 53)			
Antibodies	Negative	Borderline	Positive	Strong Positive
anti-RNP-Sm	50 (94.34%)	0 (0.00%)	3 (5.66%)	0 (0.00%)
anti-Sm	42 (79.25%)	1 (1.89%)	10 (18.87%)	0 (0.00%)
anti-SS-A (Ro-60)	42 (79.25%)	1 (1.89%)	10 (18.87%)	0 (0.00%)
anti-SS-B	44 (83.02%)	1 (1.89%)	7 (13.21%)	1 (1.89%)
anti-Ro-52	39 (73.58%)	4 (7.55%)	5 (9.43%)	5 (9.43%)
anti-Scl-70	47 (88.68%)	1 (1.89%)	5 (9.43%)	0 (0.00%)
anti-Jo-1	46 (86.79%)	1 (1.89%)	6 (11.32%)	0 (0.00%)
anti-PM-Scl100	48 (90.57%)	1 (1.89%)	4 (7.55%)	0 (0.00%)
anti-PCNA	45 (84.91%)	0 (0.00%)	8 (15.09%)	0 (0.00%)
anti-dsDNA	46 (86.79%)	0 (0.00%)	6 (11.32%)	1 (1.89%)
anti-Hi	50 (94.34%)	0 (0.00%)	2 (3.77%)	1 (1.89%)
anti-RIB	49 (92.45%)	0 (0.00%)	4 (7.55%)	0 (0.00%)
anti-Nuc	50 (94.45%)	0 (0.00%)	3 (5.66%)	0 (0.00%)

ANA—antinuclear antibodies; anti-RNP-SM—anti ribonucleoprotein/Smith antigen antibodies; anti-Sm—anti Smith antigen; anti-SS-B—anti Sjögren syndrome-related antigen B; anti-SS-A—anti Sjögren syndrome-related antigen A; anti-Ro-52—anti-ribonucleoprotein 52 kDa; anti-Scl-70—anti-DNA topoisomerase I antibody; anti-Jo-1—anti-histidyl-transfer RNA synthetase antibody; anti-PM-Scl100—anti-polymyositis-associated antigen 100 antibodies; anti-PCNA—anti-proliferating cell nuclear antigen; anti-dsDNA—anti-double-stranded DNA antibodies; anti-RIB—anti-ribosomal P protein antibodies, anti-Hi—anti-histone, anti-Nuc—anti-nuclear.

**Table 4:** Correlations between ANA titers and selected laboratory and clinical parameters in rheumatoid arthritis patients.

Correlation of Selected Parameters with ANA Titer in RA Patients (*n* = 53)		
Variable	Spearman’s R	*p*-Value
ESR	0.105	0.456
CRP	−0.028	0.844
RF	0.089	0.527
Anti-CCP	0.201	0.167
DAS28-ESR	0.112	0.424
Tender joint count	0.060	0.670
Swollen joint count	0.098	0.486

CRP—C-reactive protein, ESR—erythrocyte sedimentation rate, DAS28—disease activity score, RF—rheumatoid factor, Anti-CCP—anti-cyclic citrullinated peptide autoantibodies.

**Table 5:** Logistic analysis of predictive factors for ANA positivity in rheumatoid arthritis.

	Univariate Logistic Regression Analysis (*n* = 53)	
Parameter	OR (95% Cl)	*p*-Value
Age	1.010 (0.976–1.046)	0.5578
BMI	1.037 (0.955–1.125)	0.3866
Current smoking	1.429 (0.408–4.996)	0.5766
Duration of disease	0.968 (0.923–1.016)	0.1901
CRP	0.977 (0.920–1.039)	0.4605
ESR	0.978 (0.942–1.016)	0.2509
RF	0.999 (0.994–1.004)	0.7077
CCP	0.995 (0.988–1.003)	0.2219
Tender joint count	0.945 (0.852–1.050)	0.2931
Swollen joint count	0.901 (0.768–1.058)	0.2042
DAS28	0.770 (0.503–1.179)	0.2297

BMI—body mass index, CRP—C-reactive protein, ESR—erythrocyte sedimentation rate, DAS28—disease activity score, RF—rheumatoid factor, OR—odds ratio.

**Table 6:** Multivariate logistic regression analysis of predictive factors for ANA positivity in rheumatoid arthritis.

Predictive Values of ANA Positivity in RA (*n* = 53)		
Parameter	OR (95% Cl)	*p*-Value
Intercept	2.276 (0.569–9.101)	0.245
Duration of disease	0.955 (0.902–1.011)	0.113
CCP	0.994 (0.987–1.002)	0.148
Swollen joint count	0.913 (0.762–1.092)	0.319

CCP—anti-cyclic citrullinated peptide autoantibodies, OR—odds ratio.

## Data Availability

The raw data supporting the conclusions of this article will be made available by the authors on request.

## Supplementary Materials

The following supporting information can be downloaded at: https://www.mdpi.com/article/10.3390/antib15040058/s1, Table S1: Disease-modifying antirheumatic drugs according to ANA status in patients with rheumatoid arthritis.

## Abbreviations

The following abbreviations are used in this manuscript:

ANA	Antinuclear Antibodies
Anti-dsDNA	Double-stranded DNA antigen antibodies
Anti-CCP	Anti-Cyclic Citrullinated Peptide antibodies
Anti-Jo-1	Histidyl-tRNA synthetase antigen; anti-Centromere B antibodies
Anti-PCNA	Proliferating cell nuclear antigen antibodies
Anti-PM-Scl100	Anti-Polymyositis-Associated Antigen 100 antibodies
Anti-RNP/Sm	Anti-ribonucleoprotein/Sm complex antibodies
Anti-Ro52	Sjögren’s syndrome antigen A antibodies (Ro 52 kDa autoantigen)
Anti-Scl-70	Systemic sclerosis antigen antibodies
Anti-Sm	Smith’s antigen antibodies
Anti-SS-A	Native Sjögren’s syndrome antigen A antibodies (Ro 60 kDa autoantigen)
Anti-SS-B	Sjögren’s syndrome antigen B antibodies
Anti-Hi	Histone antigen antibodies
BMI	Body Mass Index
CRP	C-Reactive Protein
CTD	Connective Tissue Disease
DAS28	Disease Activity Score in 28 Joints
ENA	Extractable Nuclear Antigen
ESR	Erythrocyte Sedimentation Rate
EULAR	European Alliance of Associations for Rheumatology
Hep-2	Human Epithelial Type 2 Cells
IIF	Indirect Immunofluorescence
IQR	Interquartile Range
LI	Light intensity
OR	Odds Ratio
RA	Rheumatoid Arthritis
RF	Rheumatoid Factor
TEN28	Tender Joint Count (28 joints)
SW28	Swollen joint Count (28 joints)
VAS	Visual Analog Scale
