# Supplementary material for: Beyond the Surface: Antinuclear Antibodies in Rheumatoid Arthritis—Experiences from a Single-Center, Cross-Sectional Observational Study
Source: Antibodies (Basel). 2026 Jul 10;15(4):58. doi: 10.3390/antib15040058 (PMC13398195; doi:10.3390/antib15040058)
Supplement: Supplementary file 1 [file antibodies-15-00058-s001.zip › antibodies-4379011-supplementary.pdf]

Supplementary Materials: Supplementary Table S1. Disease-modifying antirheumatic drugs according to ANA status in patients with rheumatoid arthritis.

Treatment	ANA-positive (n=53)	ANA-negative (n=28)	p-value
Methotrexate	34 (64.2%)	13 (46.4%)	0.158
Biologic drugs	26 (49.1%)	12 (42.9%)	0.645
Corticosteroids	24 (45.3%)	12 (42.9%)	1.000
Leflunomide	11 (20.8%)	5 (17.9%)	1.000
Hydroxychloroquine	3 (5.7%)	4 (14.3%)	0.227
Sulfasalazine	2 (3.8%)	0 (0.0%)	0.542
